# Integrated approach to prevent functional decline in hospitalized elderly: the Prevention and Reactivation Care Program (PReCaP)

**DOI:** 10.1186/1471-2318-12-7

**Published:** 2012-03-16

**Authors:** Annemarie JBM de Vos, Kirsten JE Asmus-Szepesi, Ton JEM Bakker, Paul L de Vreede, Jeroen DH van Wijngaarden, Ewout W Steyerberg, Johan P Mackenbach, Anna P Nieboer

**Affiliations:** 1 Institute of Health Policy and Management, Erasmus University Rotterdam, P.O. Box 1738, 3000 DR Rotterdam, The Netherlands; 2 School of Population Health, The University of Western Australia, 35 Stirling Highway, 6009 Crawley, Western, Australia; 3 Department of Public Health, Erasmus Medical Centre Rotterdam, P.O. Box 2040, 3000 CA Rotterdam, The Netherlands; 4 Department R & D, Argos Zorggroep, P.O. Box 4023, 3102 GA Schiedam, The Netherlands

## Abstract

**Background:**

Hospital related functional decline in older patients is an underestimated problem. Thirty-five procent of 70-year old patients experience functional decline during hospital admission in comparison with pre-illness baseline. This percentage increases considerably with age.

**Methods/design:**

To address this issue, the Vlietland Ziekenhuis in The Netherlands has implemented an innovative program (PReCaP), aimed at reducing hospital related functional decline among elderly patients by offering interventions that are multidisciplinary, integrated and goal-oriented at the physical, social, and psychological domains of functional decline.

**Discussion:**

This paper presents a detailed description of the intervention, which incorporates five distinctive elements: (1) Early identification of elderly patients with a high risk of functional decline, and if necessary followed by the start of the reactivation treatment within 48 h after hospital admission; (2) Intensive follow-up treatment for a selected patient group at the Prevention and Reactivation Centre (PRC); (3) Availability of multidisciplinary geriatric expertise; (4) Provision of support and consultation of relevant professionals to informal caregivers; (5) Intensive follow-up throughout the entire chain of care by a casemanager with geriatric expertise. Outcome and process evaluations are ongoing and results will be published in a series of future papers.

**Trial registration:**

The Netherlands National Trial Register: NTR2317

## Background

Hospital admission is considered a health risk for older patients. Thirty-five percent of 70-year old patients experience functional decline during hospital admission in comparison with pre-illness baseline. This percentage increases to 50% for 85-year old patients [1]. Functional decline in elderly patients is not necessarily related to the medical condition of the patient. Several other factors play a major role in the occurrence of the functional decline, including iatrogenic effects of the treatment and the effects of hospitalization, such as immobilization, isolation, and inaccessibility to fluids [2]. Furthermore, age, lower functional status before hospital admission, impaired cognitive status, depression and prolonged length of hospital stay are significant predictors of hospital related functional decline in elderly patients [3-5]. Functional decline can be defined as a new loss of independence in self-care activities or as deterioration in self-care skills, measured on an activities of daily living (ADL) scale (e.g. bathing, dressing, transferring from bed to chair, using the toilet) and/or on an instrumental activities of daily living (IADL) scale (e.g. shopping, housekeeping, preparing meals) [6,7]. Not only activities of daily living can be compromised. Functional decline may also result in physical and psychosocial problems, such as dehydration, malnutrition, falls, depression, and delirium [1,2,8].

Our earlier research demonstrated that 47% of the group of elderly patients (> 60 years) can be considered to be at risk for functional decline during hospitalization, due to the presence of four or more risk factors, including home care, history of falls, polypharmacy, weight loss (more than one kilogram in the past month), and psychiatric symptoms (anxiety, depression) [4]. It is anticipated that a considerable part of these elderly patients at risk require intensive reactivation during hospital admission and after discharge in a hospital replacement care facility, often due to the patient's failure to recognize the potential problems or the lack of informal caregivers.

The literature demonstrates several approaches aimed at preventing functional decline in hospitalized elderly with mixed results. The Comprehensive Geriatric Assessment (CGA) comprising of a screening for risks for adverse outcomes, a diagnostic assessment on the presence of geriatric conditions and multidisciplinary tailored interventions, has most often been studied. Early screening of the elderly by means of the CGA has demonstrated a reduction in cognitive and functional decline in patients at risk [9,10], and to retaining quality of life and independence in activities of daily living [11]. The implementation of the CGA resulted in lower mortality rates in the elderly after six months, but not after 12 months follow-up [12]. Multidisciplinary interventions, including physical training are associated with a reduction in functional decline [13,14], reduced length of hospital stay at the same costs compared to 'regular care' [13-16], lower (re)admissions to hospital and nursing homes [17-19], reductions in fall incidence [15,20], higher perceived health and life satisfaction among patients [18,21,22]. Evidence shows that these effects are present between six and twelve months after the start of the intervention with the largest effect at three months [23]. Various studies have emphasized the importance of utilizing specialized geriatric units, often in combination with multidisciplinary follow up-treatment, including case management after hospital discharge with rehabilitation service [19,23-26].

Hospital related functional decline in elderly patients is an underestimated problem. In The Netherlands medical treatment and nursing care largely focuses on the diagnosed illness, thereby neglecting reactivation care that may prevent functional decline in the elderly patient. The Prevention and Reactivation Care Program (PReCaP) [Zorgprogramma voor Preventie en Herstel (ZPH)] was developed to address this issue by utilizing a multidisciplinary, integrated and goal-oriented approach focused at the early screening of risk factors for functional decline and the provision of a patient-oriented reactivation program. Given the large body of evidence, it is expected that this approach will lead to improved functional status and better quality of life for the elderly for twelve months after hospitalization, reductions in fall incidence, reduced length of hospital stay, lower (re)admissions to hospital and nursing homes, improved mental well-being of informal caregivers, and lower mortality [13,15,18,20-23,25,27-30].

There is a paucity of detailed descriptions of geriatric interventions in the international literature. This paper addresses this issue by presenting an outline of the Prevention and Reactivation Care Program (PReCaP) including community involvement; the roles and responsibilities of core staff; the setting and administrative structure; the care process - including identification and screening procedure, key interventions, use of the standardized Goal Attainment Scaling (GAS) method, follow-up treatment at the Prevention and Reactivation Centre, multidisciplinary approach, case management, provision of support to informal caregivers, quality assurance measures -; and the expected outcomes and benefits.

## Methods/design

### Overview

The Prevention and Reactivation Care Program (PReCaP) was developed in 2010 as a means to reduce hospital related functional decline among elderly patients by offering interventions that are multidisciplinary, integrated and goal-oriented at the physical, social, and psychological domains of functional decline. The program combines existing treatment methods and innovative care paths for reactivation into a comprehensive care package that fits the individual needs of elderly patients and their informal caregivers. In contrast to the traditional care model (Table 1), in which the reactivation treatment is provided as a separate element, the PReCaP integrates the treatment of the medical condition and the reactivation of the elderly patient. Furthermore, the PReCaP includes the following distinctive elements: (1) Early identification of elderly patients with a high risk of functional decline, and if necessary followed by the start of the reactivation treatment within 48 h after hospital admission; (2) Intensive follow-up treatment, for a maximum period of three months, of a selected patient group at the Prevention and Reactivation Centre (PRC) following referral from the multidisciplinary team. The intensive reactivation treatment is aimed at improving the patients' ability to live independently in the home environment, and is delivered concurrently with specialized nursing home care, (para) medical care, and mental health care; (3) Availability of multidisciplinary geriatric expertise during hospitalization, during admission at the PRC, and in the home environment; (4) Provision of support and consultation of relevant professionals (e.g. psychologist) to informal caregivers; (5) Intensive follow-up, for a maximum period of six months, throughout the entire chain of care (from hospital to home) by a casemanager with geriatric expertise.

**Table 1:** Differences between the Prevention and Reactivation Care Program and current geriatric Care in The Netherlands

	Prevention and Reactivation Care Program	Hospital care with follow-up care	Hospital care without follow-up care
Hospital care	Identification of vulnerable elderly patient within 48 h Assessment of risk factors for functional decline Start reactivation treatment within 48 h Clinical geriatrician Geriatric nurses	Start reactivation treatment after discharge No specific identification instrument	Start reactivation path after discharge
Hospital replacement care	Prevention and Reactivation Centre Part of treatment plan Continuation of (in hospital started) treatment focused on six domains of functional status Availability of (para)medical disciplines	Hospital replacement care Admission is patient's choice Care facility with option for treatment No structured treatment plan, but separate elements Limited number of (para)medical disciplines	Hospital replacement care not available
Home care	Geriatric care chain agreements with general practitioner and home care Case management with geriatric expertise	Follow-up care by home care organizations (not specialized in geriatrics)	Follow-up care by home care organizations (not specialized in geriatrics)
Multidisciplinary approach	Weekly multidisciplinary team meeting Treatment and care focused on medical condition and functioning in six domains (i.e. physical, mental, social, financial, home, and care) Goal-oriented approach	Key professional is responsible for treatment and interdisciplinary consults Discussion and collaboration focused on medical condition	Key professional is responsible for treatment and consults Discussion and collaboration focused on medical condition
Patient	Patient oriented integrated treatment plan Discussion treatment with patient during entire treatment path Problem solving	Separate treatment plans Treatment coherence determined by patient	Separate treatment plans Treatment coherence determined by patient
Informal caregiver	Part of treatment plan	Individual choice	Individual choice

### Community involvement

The Geriatric Network Rotterdam area [Geriatrisch Netwerk Rotterdam en Omgeving (GENERO)] is a regional geriatric network, established to improve the quality of care and wellbeing of the vulnerable elderly in the region. The PReCaP incorporates the GENERO themes 'Improvement of coordination and continuity of care and welfare' and 'Timely observation of complex problems', which are based on the needs and requirements of the elderly and their informal caregivers. Elderly stakeholders have expressed concerns about the incapability of care providers in recognizing and addressing complex geriatric problems in a timely fashion, both in primary and secondary health care. Furthermore, the elderly have indicated that they require personal and expert attention, thereby involving their social system. In addition, the elderly and informal caregivers prefer an integrated preventive care approach, and a single contact person with geriatric expertise. Given the design of the PReCaP, it is anticipated that the interventions address these issues. GENERO organizes regular network meetings, brainstorm sessions, and elderly and informal caregivers' forums to monitor the relevance of interventions, to discuss the results and to promote knowledge transfer.

### Roles and responsibilities

The Argos Zorggroep has developed and initiated the PReCaP in 2010, and is responsible for the effective implementation of the program. The ongoing consultation and support to the program is provided by the following interdisciplinary experts: nursing home physician; geriatric nurses; nurse practitioners; social workers; transfer nurses; casemanagers; and representatives from psychiatry, psychology, physiotherapy, occupational therapy, and dietetics. The specific role of each staff member is described in Table 2.

**Table 2:** Prevention and Reactivation Care Program Interventions

Intervention	PReCaP Core Staff
**Hospital**	
Identification of patient at risk within 48 h after admission	Research nurse
Assessment of risk factors for functional decline	Research nurse
Consult with patient and relatives to discuss vulnerability and risk factors	Casemanager or geriatric nurse
Biweekly Multidisciplinary Team Meeting:	Geriatrician
• Analysis of the function diagnosis in relation to the medical diagnosis	Geriatric nurse
• Design GAS care plan including advice for additional treatment aimed at functional preservation	Nurse practitioner
	Social worker
	Transfer nurse
	Casemanager
Geriatric consultation	Geriatrician
	Geriatric nurse
	Casemanager
	Transfer nurse
Interdisciplinary consultation, e.g. psychiatrist, psychologist, physiotherapist, occupational therapist, dietician, behavioral consultant	Geriatrician
	Casemanager
Support and provide treatment to informal caregiver (optional)	Social worker
Review prognosis and discharge destination (in some cases register patient at hospital replacement care facility)	Psychologist
	Geriatrician
	Geriatric nurse
	Nurse practitioner
	Social worker
	Transfer nurse
	Casemanager
Weekly telephone consultation informal caregiver	Casemanager
Hand out flyer 'PReCaP Recovery Team' to patient	Casemanager
Exit interview with patient and informal caregiver	Transfer nurse
Hand out flyer 'Prevention and Reactivation Centre' to patient (if transfer to PRC)	Transfer nurse
Handover GAS care plan to physician hospital replacement care facility	Casemanager or geriatrician
Home visit and support after hospital discharge until six months after hospital admission, including optional therapy	Casemanager
Prevention and Reactivation Centre	
Admission to PRC (including GAS care plan/medical handover)	Nurse practitioner
Review GAS care plan	Nursing home physician or nurse practitioner
Physical examination	Nursing home physician
Intake patient/informal caregiver	Nurse
Weekly Multidisciplinary Team Meeting:	Nursing home physician (coordinator)
• First MTM after one week admission PRC	Nurse practitioner Casemanager Psychiatrist (in consultation)
• Review progress and adjust GAS care plan	Social worker (in consultation)
• Casemanager home care attends MTM in week 9	Clinical geriatrician (in consultation)
Introduction and intake patient	Nurse
Treatment according to GAS care plan	Consulted disciplines
If needed additional treatment by PReCaP recovery team and other disciplines if indicated, e.g. behavioral therapist, dietician, music therapist, dance therapist, visual arts therapist	Casemanager
Hand over diary to patient (incl. therapy appointments and treatment information)	Nurse
Support with activities according to diary	Nurse
Specialized nursing home care within the socio-therapeutic environment, e.g. psychologist, physiotherapist (3 times a week), occupational therapist, speech therapist, dietician, behavioral therapist, music therapist, dance therapist, visual arts therapist, social worker	Casemanager
Review medication use	Nursing home physician
Support informal caregiver	Psychologist Casemanager
Assessment of Motor and Process Skills	Occupational therapist
Before discharge home visit (in week 9)	Occupational therapist
If needed consultation external expertise, e.g. ophthalmologist, otolaryngologist, (orthopedic) surgeon, psychiatrist, neurologist, dermatologist, rehabilitation specialist	Nursing home physician
If needed short term admission to psychiatric hospital or re-admission to hospital	Nursing home physician
Hand out flyer 'PReCaP route after discharge'	Casemanager
At discharge: write-up report GAS care plan, including advice additional treatment aimed at function preservation in the home environment	Nursing home physician (coordinator)
	Nurse practitioner Casemanager Psychiatrist (in consultation)
	Social worker (in consultation)
	Clinical geriatrician (in consultation)
At discharge: write-up discharge letter	Nursing home physician Nurse practitioner
At discharge: write-up handover	Involved disciplines
At discharge: handover care plan to general practitioner	Casemanager
If home care after PRC discharge: intake casemanager homecare in the presence of casemanager PReCaP ('warm handover')	Casemanager

Implementation of the PReCaP will require an increased pro-active and methodological approach from involved staff due to the preventive and systematic nature of the program. Given the patient oriented approach, it is expected that the implementation of the program will lead to increased collaboration between the involved disciplines and departments, and a possible shift in existing roles and responsibilities. For example, informal caregiver support will be provided by the social worker, the psychologist or the casemanager depending on the individual situation and existing relationships.

### Setting and administrative structure

Since 2010, the PReCaP interventions have been implemented in the Vlietland Ziekenhuis, Schiedam, a 450-bed regional teaching hospital, serving a large community as well as a referral population. The hospital has a collaborative agreement with the Argos Zorggroep regarding patient transfer to the PRC at the DrieMaasStede Nursing and Reactivation Centre, and collaborative liaisons with primary care providers.

The administrative and decision making body of the PReCaP consists of a working group within the hospital and the PRC, and includes the program director/psycho geriatrician, program leader, casemanagers, and geriatric nurses. The working groups meets monthly to set goals and priorities for the program; establish program procedures and guidelines; monitor progress; address problems; and reach consensus on intervention issues.

An Implementation Taskforce (ITF) was established in 2009 to provide expert advice on the design and development of the PReCaP, to facilitate knowledge transfer, and promote the broad implementation of the PReCaP results in the chain of care for the elderly in the region as well as further afield in the future. During the development phase, the ITF acted as a sounding board for the PReCaP team, which assisted, for example in the decision-making process regarding the implementation of the program in different settings. In addition, the ITF advised in the assessment of the applicability of developed indicators to map out the care process, and on the contents and quality of the geriatric nurses training program. Following the evaluation phase in 2012, it is anticipated that the ITF will develop an implementation plan for other geriatric care settings based on the result of the PReCaP program. The ITF meets four times per year, and consists of 15 members, represented by geriatricians, nursing home physicians, geriatric nurses, psychiatrists, rehabilitation specialists, general practitioners, patient council representatives, home care providers, and health insurance representatives. The composition of the ITF in terms of which members will be represented, depends on the phase of the PReCaP, the specific parts of the implementation, and the results to be discussed.

### Process of care

#### Identification and screening procedure

Every patient of 65 years or older, admitted to the Vlietland Ziekenhuis for at least two days, is screened, within 48 h after admission, to identify those at risk for hospital related functional decline. In order to pre-test the developed identification- and screening methods, we conducted a pilot study in the Vlietland Ziekenhuis, which involved 296 patients and 160 informal caregivers. Based on the results of the pilot, we have selected the following two-step triage:

1. The Identification of Seniors at Risk - Hospitalized Patients (ISAR-HP), a validated four-item instrument to predict functional decline during hospital admission [31,32]. The instrument is administered to patients of 65 years or older, who are expected to be admitted to hospital for more than 48 h. We have set the inclusion cut-off score at ≥ 1, in contrast to the cut-off score of ≥ 2 as proposed by Buurman et al. [31] to be as inclusive as possible, while ensuring the inclusion of patients with at least one risk factor. Exclusion criteria are the inability to answer questions or to follow instructions, due to cognitive problems (Mini Mental State Examination (MMSE) score < 12 [33]), the inability to understand the Dutch language, or a life expectancy of less than three months.

2. The NeuroPsychiatric Index (NPI-Q) and the Mini Mental State Examination (MMSE). The NPI-Q is a validated short version of the Neuropsychiatric Index, which aims to identify neuropsychiatric symptoms in the last month, including aggression, delusions, and hallucinations [34]. The NPI-Q will be administered by means of a telephone interview with the informal caregiver, and aims to identify eligible patients for admission to the PRC, and to measure the emotional burden for the informal caregiver. The MMSE aims to measure cognitive functioning via interview questions related to: orientation in time and place; short-term and middle-term memory; comprehension; and additional cognitive dimensions [33,35]. Based on the results from the pilot study, the inclusion criteria for admission to the PRC are set at either an ISAR-HP score of ≥ 2, a NPI-Q score of ≥ 3, or a MMSE score of > 12 and ≤ 27 to ensure inclusion of patients who will most benefit from treatment at the PRC.

Following the two-step triage written informed consent for participation in the study is obtained from participants.

#### Key interventions

The PReCaP interventions are presented in Table 2 and include a description of the core staff that is required to carry out the particular intervention. The identification and screening procedure is described above. Key interventions carried out by the PReCaP core staff include: biweekly multidisciplinary team meetings; design of the GAS care plan (see Goal Attainment Scaling); interdisciplinary consultation (psychiatrist, psychologist, physiotherapist, occupational therapist, dietician, behavioral consultant); case management; provision of support and treatment for the informal caregiver; and review of the prognosis and discharge destination.

#### Follow-up treatment at the prevention and reactivation centre

A specific part of the PReCaP entails the intensive reactivation treatment at the Prevention and Reactivation Centre (PRC) after hospital discharge, which is aimed at improving the patients' ability to live independently in the home environment. Therefore, the PRC provides specialized nursing home care in combination with intensive theme oriented reactivation treatment, paramedical treatment (e.g. physiotherapy, dietetics, occupational therapy); psychiatric treatment (including short term admission in a psychiatric hospital or a psycho-geriatric reactivation unit if necessary), and support and psychotherapy sessions for informal caregivers if required (Table 2). The PRC treatment is novel in The Netherlands, since there are no facilities that offer this type of intensive reactivation treatment for frail elderly people with complex health problems. In order to maximize continuity, case management during hospital and PRC admission is executed by the same nurse with geriatric expertise. The multidisciplinary team, consisting of the nursing home physician (coordinator), nurse practitioner, casemanager, and (if consulted) paramedical professionals, psychologist, social worker and clinical geriatrician, convenes weekly. During these meetings, the team accesses the patient's data in the online GAS data base (see Goal Attainment Scaling), reviews and discusses the patient's progress, and adjusts the GAS care plan accordingly (after consulting the patient and the informal caregiver). The maximum admission period at the PRC is three months. On discharge from the PRC, the multidisciplinary team designs a care plan for the home setting, which contains advice on further treatment or support, and recommendations for specific health care providers. The casemanager is responsible for the care plan handover to the general practitioner, and liaises with primary care professionals and institutions about the implementation of the care plan. Given the large body of evidence, it is expected that the intensive reactivation treatment at the PRC will lead to improved functional status and better quality of life for the elderly [13,15,18,20-23,25,27-30]. Based on earlier research, we estimate that 10-20% of the patients of 65 years or older will benefit from the intensive reactivation program at the PRC (unpublished data).

#### Additional follow-up treatment routes

Depending on the patient's requirements and bed availability, five additional follow-up routes for reactivation within the PReCaP are available: (1) Reactivation at a Cerebral Vascular Accident (CVA) unit, somatic reactivation unit or psycho-geriatric unit; (2) Day treatment at a somatic unit, psycho-geriatric unit or day treatment unit; (3) Admission to a retirement home; (4) Admission to a nursing home; and (5) Treatment at home. Regardless of the selected follow-up route, the PReCaP casemanager coordinates the patient's care and monitors the patient's and informal caregiver's progress according to the GAS care plan. Additional disciplines can be consulted if necessary, e.g. occupational therapist, speech therapist, dietician, behavioral therapist, music therapist, psychomotor therapist, visual arts therapist, or social worker. If the patient receives treatment in the home setting, the casemanager visits the patient monthly up until six months after the hospital admission date.

#### Goal attainment scaling

The Goal Attainment Scaling (GAS) is used to evaluate complex interventions in frail elderly patients by means of facilitating the individualization of patients' goals according to their needs [36-39]. Bakker et al. [40,41] developed a modified version of the GAS by standardizing the measurement through the application of a summary formula that calculates the extent to which the patients' goals are met. Within 48 h after admission, the patient's functional state, varying from totally functional dependent to independent, is scored for the six domains of functional decline: somatic, cognition, personality, emotional and rational experiences, social environment, and life history and/or trauma (Table 3). Simultaneously, a goal GAS-score of 1 or 2 points higher is determined for each domain of functional decline. In this way, the GAS assists in formulating individual goals, developing a personalized treatment plan, monitoring both the patient's and informal caregiver's progress, and adjusting the interventions in a timely manner as necessary.

**Table 3:** Scoring Goal Attainment Scaling

Domain	Functional State Score				
	Totally functionally dependent (1-2)	Regularly functionally dependent (3-4)	No help needed, only guidance (5)	Functionally independent with adjustments and/or aids (6)	Independent (7)
Somatic					
Cognition					
Personality					
Emotional and rational experiences					
Social environment					
Life history and/or trauma					

#### Multidisciplinary approach

The PReCaP incorporates an integrated program of interventions combining different elements of care that are offered by a multidisciplinary team with geriatric expertise, including (but not limited to) geriatrician, geriatric nurse, nurse practitioner, social worker, transfer nurse, and casemanager (Table 2). It is anticipated that this approach will lead to improved functional status, reductions in fall incidence, reduced length of hospital stay, lower (re)admissions to hospital and nursing homes, improved mental well-being of informal caregivers, and lower mortality [13,15,18,20-23,25,27-30].

Hospital and primary services are fully integrated in the PReCaP. Working agreements have been reached between and within the first line (e.g. general practitioner, home care organizations) and second line health care organizations (e.g. hospital, PRC). These agreements are considered important for an efficient and timely care process, and include referrals between primary and secondary health care; paramedic consultations during the hospital and PRC phase; and consultations between the general practitioner, home care, social work, paramedics (e.g. physiotherapy), municipality (in order to prevent long waiting lists for medical aids). The casemanager coordinates alignment between hospital, PRC, general practitioner, and home care in the implementation of these agreements. The involved disciplines meet twice a week during the Multidisciplinary Team Meeting (MTM) to discuss new patients, to develop individual treatment plans and to evaluate the current patients' progress.

#### Case management

The casemanager with geriatric expertise acts as the patient's casemanager throughout the entire chain of care, i.e. hospital care, hospital replacement care, and primary care until six months after hospital admission. In consultation with the PReCaP team and (in the home situation) primary health care providers, the casemanager coordinates the multidisciplinary care process, supports and motivates the patient in treatment adherence, and monitors the patient's risk factors for functional decline throughout the reactivation period. In other words, the casemanager is the patient's broker to ensure the most appropriate form of health care, as well as a provider of the treatment. The specific casemanager's tasks are:

■ To ensure follow-through of the treatment plan, which will be handed over to the general practitioner after hospital discharge;

■ To establish the follow-up multidisciplinary primary care team in consultation with the general practitioner;

■ To include home care in the multidisciplinary team;

■ To maintain contact with representatives of the social support system and welfare organizations;

■ To visit the patient and informal caregiver at home. The first visit takes place within two weeks after hospital or PRC discharge, followed by monthly visits (or more frequently if necessary) until six months after the hospital admission date;

■ To motivate and provide support to the patient and informal caregiver in adhering to the treatment plan;

■ To monitor the presence of risk factors for functional decline, e.g. use of medicines, weight, functioning of the informal caregiver;

■ To liaise with the general practitioner, the multidisciplinary team, the hospital, and the PRC.

Although casemanagement has been valued for improving access to health care, increasing psychosocial support and improving communication with health professionals, it may not change overall hospital admissions due to increased case-finding [42].

#### Provision of support to informal caregivers

The GAS incorporates the social environment, including the social activities and informal care system in the evaluation of the risk factors for functional decline and the targeted interventions. Informal caregiver support may not directly influence the patient's social environment, yet it is expected to increase the resources of the patient's social environment. This may include providing guidance and information, as well as the opportunity to consult and receive treatment from relevant professionals (e.g. psychologist), aimed at reducing the burden on the informal caregiver [41].

#### Quality assurance measures

Before the start of the PReCaP, the Vlietland Ziekenhuis and ArgosZorggroep have developed an education program to train geriatric nurses and nurse practitioners. To date, 50 geriatric nurses have been trained to work in the hospital, at the PRC, or in the home care in order to ensure a streamlined chain of care in the PReCaP. Furthermore, the hospital working group, including program director/geriatrician, program leader, casemanagers, and geriatric nurses convenes monthly to discuss the implementation and quality of the intervention and to address implementation issues if necessary.

### Evaluation

The PReCaP will be evaluated to determine the extent to which the PReCaP leads to improved geriatric care, which is cost-effective in comparison to current geriatric care in The Netherlands. The evaluation objectives are:

▪ To determine the validity of the PReCaP screening instruments;

▪ To identify the extent to which the PReCaP leads to the prevention of functional decline in elderly patients and improved quality of life for informal caregivers;

▪ To determine the contribution of the treatment at the PRC to overall effectiveness of the PReCaP;

▪ To determine the extent to which the PReCaP leads to an improved structure and process of care in comparison to current geriatric care in The Netherlands (in particular with regard to the content of care, patient logistics and information logistics); and

▪ To quantify the cost-effectiveness of the PReCaP in comparison to current geriatric care in The Netherlands.

The evaluation will require a concurrent mixed methods design, in which a combination of qualitative and quantitative research methods will be used. Empiric evidence regarding the immediate effects of the PReCaP, including functional status and quality of life for the elderly and the informal caregiver, as well as data regarding the process aspects of the PReCaP will be collected. The latter may include modification of the intervention over time or a description of the contextual factors influencing the intervention effectiveness. A quasi-experimental research design will be used to evaluate the overall PReCaP, in which the impact on functional status and quality of life of the elderly will be measured in a prospective cohort study. The specific PRC component will be evaluated using a randomized controlled trial design [43].

Three Dutch hospitals with different levels of geriatric care will participate in the evaluation study:

1) Vlietland Ziekenhuis, Schiedam, a 450-bed regional hospital with a geriatric department, hospital replacement care (PRC), and provisions for follow-up in primary care;

2) Sint Franciscus Gasthuis Rotterdam, a 613-bed teaching hospital with hospital replacement care (Zorg Hotel Aafje), but without a clinical geriatric department or provisions for follow-up in primary care; and

3) Ruwaard van Putten Ziekenhuis, Spijkenisse, a 288-bed regional teaching hospital without a geriatric department, hospital replacement care, or provisions for follow-up in primary care.

These hospitals have been selected, due to comparable patient case mix and different levels of geriatric care. The PReCaP is offered in the Vlietland Ziekenhuis (intervention setting). Conventional care ('care as ususal') is offered in the control setting of the Sint Franciscus Gasthuis and the Ruwaard van Putten Ziekenhuis. Given that hospital replacement care is a common type of geriatric care in The Netherlands, the option for admission to an external hospital replacement care facility after hospital discharge will be offered to the elderly patients in the two control settings.

#### Power calculation

Based on the average number of elderly patients admitted to the three hospitals annually, a sample of 1100 patients will be included in the intervention hospital (including 200 patients in the PRC). A sample of 500 patients will be included in both control hospitals. Based on the pilot results (Katz-15 ADL score), it is expected that a baseline population of n = 1100 in the intervention hospital will result in approximately 700 patients analyzable at three months, and a group of 500 in the control hospitals will result in 300 patients analyzable at three months. Using an effect size of 0.25 will produce statistical power of 95%.

The study protocol was approved by the Medical Ethics Committee of the Erasmus Medical Centre, Rotterdam, The Netherlands under protocol number MEC2011-041.

#### Effect evaluation

The effect evaluation will measure the primary outcome data regarding physical functioning, functional decline risk factors, quality of life, and experienced informal caregiver burden at three points in time, i.e. (1) at admission; (2) at three months after admission; and (3) at twelve months after admission.

#### Process evaluation

The process evaluation will measure the extent to which the PReCaP leads to a better structure and process of care, in comparison with current forms of geriatric care in The Netherlands. This involves the coordination of different forms of care, patient logistics, information logistics and support. Improving coordination between care providers and the integrated care provision for the elderly and their informal caregivers is expected to result in improved outcomes. Process data will be collected by utilizing a set of process indicators in order to objectively assess the impact of the implementation of the program [43].

#### Intervention fidelity

The intervention fidelity will be measured to determine the adherence to the PReCaP protocol. Fidelity measurement is essential in order to maintain internal validity and to ensure a fair comparison of the results between the intervention and control settings. Results without a fidelity check may be due to an effective intervention or contamination from other interventions [44]. The issue of intervention fidelity also pertains to external validity. In order for a particular intervention to be adopted by other hospital settings, sufficient information about the method, fidelity, and effectiveness is essential [45-47].

The evaluation study commenced in March 2010, and allowing for the twelve month follow-up measurements, is expected to be completed by June 2012.

## Discussion

Thirty-five percent of patients aged over 70 years function less well after hospital discharge compared to before hospital admission. Despite the high prevalence of predictors of functional decline, this percentage increases to 65% for patients aged 90 years and older with only 20% of the functional decline related to the hospital diagnosis [1]. To date, geriatric hospital care in The Netherlands focuses on the medical treatment with less attention for reactivation care aimed at preventing functional decline in the hospitalized elderly. Furthermore, elderly patients are largely left to their own devices after hospital discharge. In order to retain the ability to cope and enjoy a quality of life, reactivation care should be organized concurrently with the medical treatment, and commence as early as possible after hospital admission and continue well after hospital discharge in a multidisciplinary harmonized fashion [9,10,48].

This paper describes the Prevention and Reactivation Care Program (PReCaP), which incorporates a package of interventions aimed at retaining the elderly patient's function during and after hospital admission. The program starts within 48 h after hospital admission, and includes an integrated individual treatment plan based on the physical, mental and social domains of functional decline. Furthermore, hospital reactivation treatment is followed by intensive reactivating care in the Prevention and Reactivation Centre for a selected group of elderly patients. After this intensive period, further treatment and support takes place in primary care for up to six months after the hospital admission date. A casemanager with geriatric expertise coordinates the multidisciplinary care plan in close collaboration with the general practitioner and home care; supports and motivates the elderly patient and informal caregiver to adhere to the care plan; and monitors the risk factors for functional decline in the home situation.

The PReCaP interventions are implemented in the Vlietland Ziekenhuis, Schiedam, The Netherlands since November 2010. Given the multidisciplinary approach and the complexity of the PReCaP interventions, it is highly likely that deviations from the protocol will occur in daily practice. Therefore, an intervention fidelity study will be carried out to measure the extent to which the interventions are implemented according to the protocol. Moreover, it is anticipated that fidelity measurement will yield results regarding the barriers and enabling factors for adherence to the protocol. These results in turn, will assist in further refining the PReCaP and adapting the program for other hospital settings where elderly patients at risk for functional decline can benefit from the PReCaP interventions and philosophy.

## Abbreviations

PReCaP: Prevention and Reactivation Care Program; PRC: Prevention and Reactivation Centre; ADL: Activities of daily living; IADL: Instrumental activities of daily living; CGA: Comprehensive Geriatric Assessment; GAS: Goal Attainment Scaling; GENERO: Geriatric Network Rotterdam area [Geriatrisch Netwerk Rotterdam en Omgeving]; ITF: Implementation Taskforce; ISAR-HP: Identification of Seniors at Risk - Hospitalized Patients; MMSE: Mini Mental State Examination; NPI-Q: NeuroPsychiatric Index.

## Competing interests

The authors declare that they have no competing interests.

## Authors' contributions

AJBMdV prepared the manuscript and revised it for important intellectual content. KJEA-S drafted the manuscript and revised it for important intellectual content. TJEMB designed the intervention protocol and revised the manuscript for important intellectual content. PLdV revised the manuscript for important intellectual content. JDHvW participated in the design of the evaluation study and revised the manuscript for important intellectual content. EWS participated in the design of the evaluation study and revised the manuscript for important intellectual content. JPM participated in the design of the evaluation study and revised the manuscript for important intellectual content. APN participated in the design of the evaluation study and revised the manuscript for important intellectual content. All authors approved final version of the manuscript to be published.

## Pre-publication history

The pre-publication history for this paper can be accessed here:

http://www.biomedcentral.com/1471-2318/12/7/prepub
